# The development of stretchable and self-repairing materials applied to electronic skin

**DOI:** 10.3389/fchem.2023.1198067

**Published:** 2023-04-28

**Authors:** Mei Li, Chuanqi Miao, Muhua Zou, Jiahu Guo, Hongzhen Wang, Miao Gao, Haichang Zhang, Zhifeng Deng

**Affiliations:** ^1^ National and Local Joint Engineering Laboratory for Slag Comprehensive Utilization and Environmental Technology, School of Materials Science and Engineering, Shaanxi University of Technology (SNUT), Hanzhong, Shaanxi, China; ^2^ Key Laboratory of Rubber–Plastic of Ministry of Education (QUST), School of Polymer Science and Engineering, Qingdao University of Science and Technology, Qingdao, China; ^3^ CART Tire Co., Ltd, Qilu SEZ, Krong Svay Rieng, Svay Rieng, Cambodia

**Keywords:** electronic skin, flexible electronic devices, stretchable conductors, self-healing conductors, organic semiconductors

## Abstract

Flexible electronic devices play a key role in the fields of flexible batteries, electronic skins, and flexible displays, which have attracted more and more attention in the past few years. Among them, the application areas of electronic skin in new energy, artificial intelligence, and other high-tech applications are increasing. Semiconductors are an indispensable part of electronic skin components. The design of semiconductor structure not only needs to maintain good carrier mobility, but also considers extensibility and self-healing capability, which is always a challenging work. Though flexible electronic devices are important for our daily life, the research on this topic is quite rare in the past few years. In this work, the recently published work regarding to stretchable semiconductors as well as self-healing conductors are reviewed. In addition, the current shortcomings, future challenges as well as an outlook of this technology are discussed. The final goal is to outline a theoretical framework for the design of high-performance flexible electronic devices that can at the same time address their commercialization challenges.

## 1 Introduction

After years of research and development, electronic products based on organic materials have made significant progress in performance, stability, and production costs compared to traditional materials, such as organic field-effect transistors (OFET) (Vissenberg et al., 1998; Meijer et al., 2003; Payne et al., 2005; Muccini, 2006; Wang et al., 2012; He et al., 2014), organic photovoltaic cells (OPVs), and organic solar cells (OSCs) (Siebbeles, 2010; Gruverman et al., 2011; Zhang et al., 2012; Bredas, 2014; Cnops et al., 2014; Tumbleston et al., 2014). With the efforts of scientific researchers, the above products have achieved good practical use efficiency and have great commercial prospects. Most organic electronic devices are assembled from several components, which ensure that the device exhibits good functionality and performance. For example, OFET is typically composed of gate electrodes, drain electrodes, organic semiconductor layers, insulating layers, and grid electrode. Although these components provide performance, these materials typically have great rigidity, which resulting in the inflexibility of electronic devices, and limits the development of stretchable and skin like electronic products (Shcherbina et al., 2017; Etiwy et al., 2019; Bent et al., 2020). With the progress of the organic electronics industry, the research of flexible electronic materials is also advancing.

The emergence of electronic skin (flexible electronic devices) represents the phased significance of stretchable, flexible, and dexterous electronic products. Electronic skin, also known as a new wearable flexible bionic tactile sensor, is a new type of electronic material that can simulate human skin and provide biocompatibility. The simple structure of electronic skin can be processed into various shapes, which attracting increasing attention. To simulate the good performance of human skin during actual use, corresponding materials need to have a certain degree of flexibility and self-healing ability (Kim et al., 2011; Jeong et al., 2012). So far, some work has been done on stretchable and self-healing organic electronic devices, and significant progress has been made (Liang and Stephen, 2010; Kuribara et al., 2012; Hammock et al., 2013; Bauer et al., 2014; Kim et al., 2014; Son et al., 2014; Xie and Wei, 2014; Kim and Lee, 2015; Minev et al., 2015; Larson et al., 2016). In this review, we summarize the methods used to develop stretchable and self-healing materials and their synthetic devices.

## 2 Stretchable semiconductors

**FIGURE 1:**
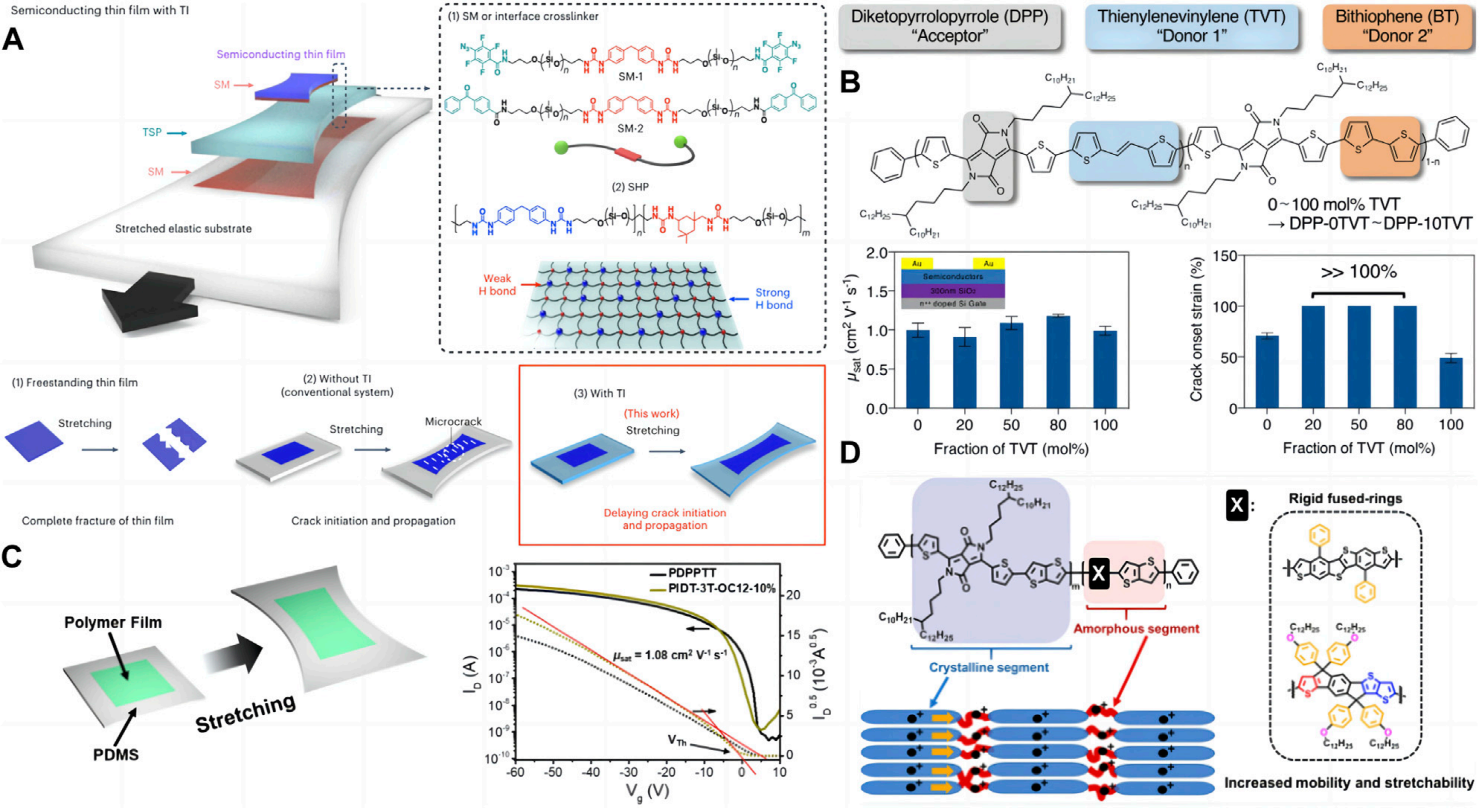
**(A)** Schematic of a TI between a semiconducting film and an elastic substrate. The TI bonding is enabled by two essential chemical components: (1) SM and (2) siloxane-based SHP capable of self-recoverable energy dissipation. A TSP is composed of a mixture of 90 wt% SHP as the energy-dissipating matrix and 10 wt% SM as the crosslinker. Schematic of the fracture conditions of a polymer thin film under various conditions. Under the freestanding condition (1), a polymer thin film undergoes brittle fracture separated into two pieces by stretching. When the thin film is attached onto an elastic polymer substrate (2), microcracks form in the polymer thin film instead of complete fracture. This work shows that embedding a TI layer delay crack initiation and propagation (3) (Kang et al., 2022). **(B)** Chemical structure of terpolymers used in this study. Based on the TVT unit fraction, terpolymers were named as DPP-0TVT (0 mol% TVT) ∼ DPP-10TVT (100 mol% TVT). Mobility of the terpolymers measured from top-contact-bottom-gate transistors given in the top left corner (measured in air). Error bars represent SD. Crack onset strain of the terpolymers. Our terpolymers showed tremendous crack onset strain >100% strain. Error bars represent SD (Mun et al., 2021). **(C)** Design of intrinsically stretchable semiconducting polymers (Liu et al., 2021). **(D)** Scheme illustrating how the polymer film is deformed on the supported PDMS substrate. Transfer curves of spin-coated polymer thin films (PDPPTT and PIDT-3T-OC12-10%) in rigid transistor configuration (the red lines are fitted for mobility and threshold voltage) (Liu et al., 2021).

The flexible function of some electronic devices is necessary, for example, sometimes it is necessary to attach electronic materials to surfaces with complex shapes, which requires materials to have good flexibility to adapt to different usage scenarios. Compared to traditional inorganic crystalline materials (such as silicon), most polymer materials are inherently more flexible, and semiconductor polymer films are the basic elements of soft electronic products for wearable and biomedical applications (Hsin-Chiao et al., 2021; Dimov et al., 2022; Mei, 2022). However, semiconductor polymers with high mobility are often brittle and prone to brittle fracture under small strains, which limits their application in electronic devices. Recently, Bao’s team (Kang et al., 2022) constructed a dynamic noncovalent cross-linked network Tl bond between semiconductor thin films and substrates (Figure 1A), which allows for high interfacial toughness between layers, inhibits delamination and strain delocalization, and enables crack initiation and propagation to occur significantly at higher strains. Specifically, the crack strain of high mobility semiconductor polymer films has increased from 30% to 110%, and there are no significant microcracks. In other semiconductor polymers, the Tl strategy changes the thin film from brittle fracture to ductile fracture (Wang et al., 2017), greatly improving the flexibility of the material. During the design process of organic semiconductor materials, their mechanical properties and carrier mobility are inversely proportional (Oh et al., 2016; Zheng et al., 2020), which means that the improvement of tensile properties will reduce carrier mobility. Bao et al. (Mun et al., 2021) developed a trimer based stretchable polymer semiconductor (Figure 1B) (taking DPP, TVT, and BT terpolymers as examples). The ternary polymers maintain short-range aggregation, reducing the overall crystallinity and average crystal domain size, compared to conventional polymer structures with only one comonomer unit, the resulting polymer maintains a high mobility (>1 cm^2^·V^−1^·s^−1^), while having better extensibility (>100% strain) and mechanical reversibility (Figure 1B).

To increase the flexibility of semiconductor materials, flexible conjugated destruction groups can be introduced into the conjugated backbone of semiconductor polymers (Zhao et al., 2015; Savagatrup et al., 2016; Galuska et al., 2020). These groups can reduce the overall rigidity of the polymer skeleton and the crystallinity of the polymer film, thereby improving tensile properties. However, most of these groups are not conjugated, which structure can have a negative impact on the transfer of carriers in the conjugated polymer skeleton, and would reduce the electrical properties of organic semiconductor materials. To solve this problem, starting from the side group is an effective strategy. Liu et al. (2021) introduced rigid conjugated side groups into the polymer skeleton. Firstly, this rigid side group can induce the formation of amorphous domains and help to improve tensile properties (Figure 1C). Secondly, this side group is conjugated, which can still keep the polymer main chain conjugated, the rigidity and coplanarity of the side groups also facilitate the transport of carriers on the polymer skeleton. In addition, the *π* conjugated extended molecular structure can increase the probability of overlapping π-π orbitals between molecules, thereby facilitating charge transfer between molecular chains and improving carrier mobility. In fully stretchable transistors, the polymer exhibits a mobility of 0.27 cm^2^·V^−1^·s^−1^ at 75% strain and maintains its mobility after undergoing hundreds of stretch release cycles at 25% strain (Figure 1D). So far, researchers have reported some rigid fusion ring molecules with excellent electrical properties (Hideaki et al., 2007; Hakan et al., 2009; Kazuki et al., 2011; Toan et al., 2012; Zhang et al., 2010; Wei et al., 2013; Jeong-Il et al., 2015; Yamamoto et al., 2017; Okamoto et al., 2020).

## 3 Self-healing conductors

**FIGURE 2:**
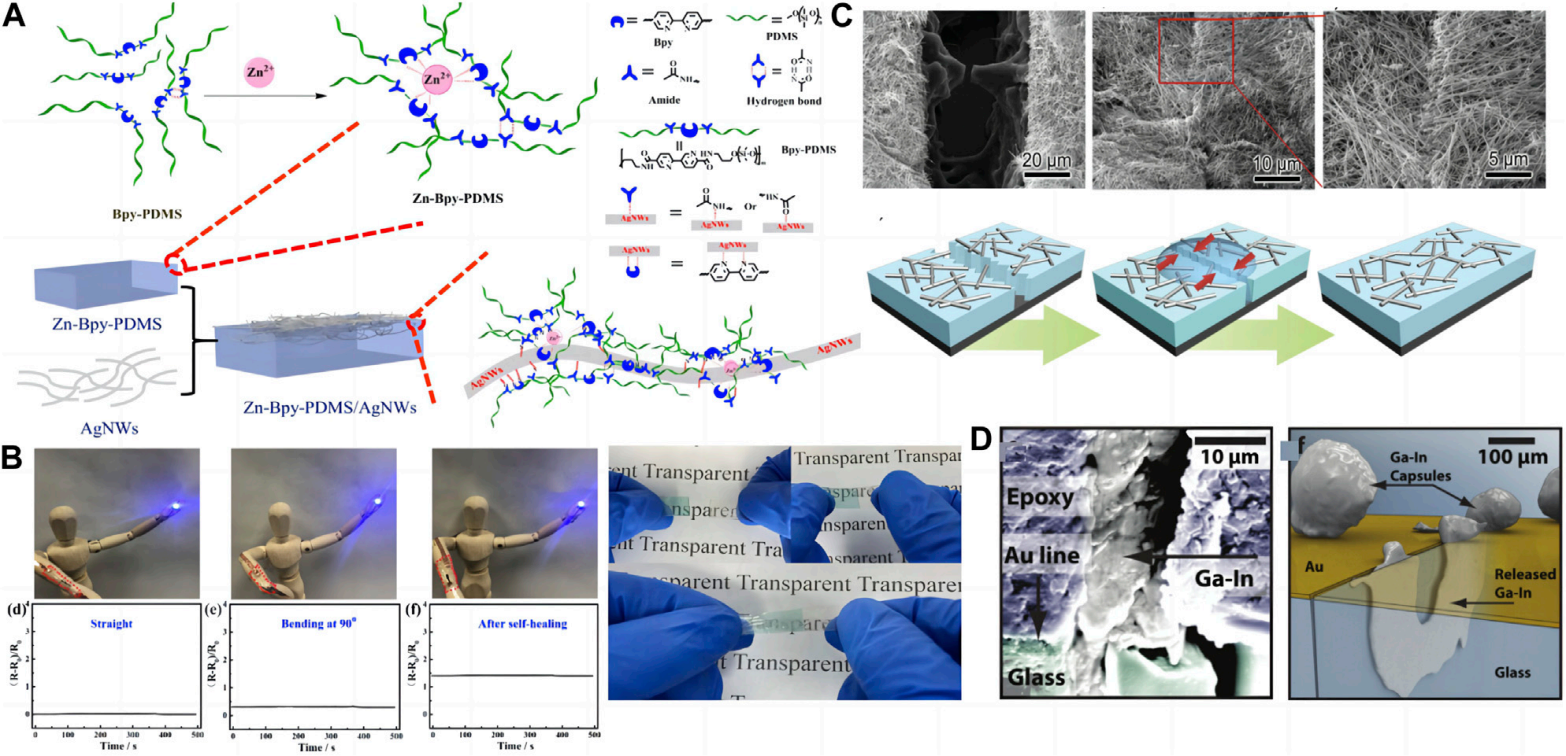
**(A)** Fabrication of the transparent skin-like conductor. Schematic structure of the self-healing Zn-Bpy-PDMS polymer and the interfacial bonding of the Zn-Bpy-PDMS/AgNWs conductor (Ye et al., 2019). **(B)** Resistance change test of materials after self-repair Photos to demonstrate the self-healing capability of Zn-Bpy-PDMS: cut, self-healing (herein, 24 h at room temperature), and stretching after healing (offside) (Ye et al., 2019). **(C)** Electron microscope images and schematic diagrams before and after conductor self-healing (Li et al., 2012). **(D)** Schematic diagram of the crack plane where the microcapsule and liquid metal have been released to the healing specimen (Blaiszik et al., 2011).

Human skin can face very complex situations in practice. In addition to being stretchable, being able to self-repair when injured is the most important ability. To better simulate human skin, electronic skin needs to have the ability to self-repair to face possible damage during actual use. To improve the lifespan and working conditions of electronic skin, and as people become increasingly interested in self-repair materials, developing its self-healing ability is the key to the next-generation of electronic skin. Hence, this article will provide an overview of the development of self-healing conductors for electronic skin. Some progress has been made in the study of artificial mechanical self-healing. Cordier et al. (2008) demonstrated the first room temperature self-healing elastomer by combining hydrogen bonds into a polymer matrix. Wudi et al. developed a plastic that can self-repair under thermal action using the dynamic covalent bond between furan and maleimide (Chen, 2002). However, most of these polymers are insulated. To obtain an ideal electronic skin, it is necessary to combine the self-healing ability and conductivity of the material. Introducing dynamic reversible bonds into conductive polymers is the most direct method for manufacturing self-healing conductors (Williams et al., 2007). In addition, combining conductive materials with polymer substrates that provide self-healing ability is an effective strategy, which is like the method for preparing stretchable conductors. Ye et al. (2019) prepared a transparent, stretchable, and self-healing conductor (Figure 2A) by semi embedding silver nanowires (AgNWs) into an elastic substrate based on PDMS. The elastomer was modified with a bipyridine (Bpy) ligand and further crosslinked by adding Zn^2+^ as a coordination agent (Zn-Bpy-PDMS). This material exhibits excellent electrical conductivity (76.2 Ω/sq) and retains its properties significantly after self-healing at room temperature (Figure 2B). Due to the Ag-N dynamic bonding formed between the conductive material and the elastic substrate, the diffusion of AgNWs is increased and the performance is further improved. Similarly, Li et al. (2012) demonstrated a simple method for producing a highly conductive film with self-healing ability by depositing an AgNW layer on a heatable bPEI/PAA-HA film (Figure 2C). After adding deionized water, the PEM film will expand and promote the healing of the fractured surface during recombination of dynamic ionic bonds. In addition, hydrogen bond interactions occur between the carboxylic acid groups on the surface of bPEI/PAA-HA films and the pyrrolidone groups of PVPON modified AgNWs, and this strong interaction drives the movement and contact of the conductive layer, helping to restore conductivity. In addition, it is a novel method to prepare self-healing conductors by encapsulating conductive substances. White’s team has developed two materials, one is a self-healing conductor with charge transfer salt capsules. Polyurea formaldehyde core shell microcapsules are respectively loaded into TTF-TCNQ solutions in different solvents (Odom et al., 2010). The mixed capsule is then plated into the gap between the gold electrodes. After mechanical damage, the polymer capsule ruptures, and the TTF and TCNQ components are mixed to form a conductive charge transfer salt to fill the gap, thereby restoring conductivity. Using a similar method, Blaiszik’s group (Blaiszik et al., 2011) developed another self-healing conductor using EGaIn as a therapeutic agent (Figure 2D). Compared to the previous method, liquid metal does not require mixing, simplifying the preparation process.

Obviously, the strategy of capsule repair agents is effective and fast, but with the consumption of the number of capsules in the same region, the repetitive self-healing ability of the same region will be limited, which requires further exploration.

## 4 Conclusion

In summary, this review focuses on the development and design strategies of stretchable and self-healing conductors in electronic skin, while the development of intrinsically stretchable conductors is relatively blank.

Based on the current progress in artificial intelligence technology and the concept of sustainable and environmentally friendly materials, developing biodegradable flexible devices is a good idea. In addition, the demand for flexible electronic devices such as electronic skin in the pharmaceutical, aerospace, and new energy industries will increase day by day, making it a promising field in the future.

## References

[B1] Bauer S. Bauer-Gogonea S. Graz I. Kaltenbrunner M. Keplinger C. Schwödiauer R. (2014). 25th anniversary article: A soft future: From robots and sensor skin to energy harvesters. Adv Mater. 26 (1), 149–162. 10.1002/adma.201303349 24307641 PMC4240516

[B2] Bent B. Goldstein B. A. Kibbe W. A. Dunn J. P. (2020). Investigating sources of inaccuracy in wearable optical heart rate sensors. npj Digit. Med. 3 (1), 18. 10.1038/s41746-020-0226-6 32047863 PMC7010823

[B3] Blaiszik B. J. Kramer S. L. B. Grady M. E. McIlroy D. A. Moore J. S. Sottos N. R. (2011). Autonomic restoration of electrical conductivity. Adv. Mater. 24 (3), 398–401. 10.1002/adma.201102888 22183927

[B4] Bredas J.-L. (2014). When electrons leave holes in organic solar cells. Science 343 (6170), 492–493. 10.1126/science.1249230 24482470

[B5] Chen X. Dam M. A. Ono K. Mal A. Shen H. Nutt S. R. (2002). A thermally Re-mendable cross-linked polymeric material. Material. Sci. 295 (5560), 1698–1702. 10.1126/science.1065879 11872836

[B6] Cnops K. Rand B. P. Cheyns D. Verreet B. Empl M. A. Heremans P. (2014). 8.4% efficient fullerene-free organic solar cells exploiting long-range exciton energy transfer. Nat. Commun. 5, 3406. 10.1038/ncomms4406 24603622

[B7] Cordier P. Tournilhac F. Soulié-Ziakovic C. Leibler L. (2008). Self-healing and thermoreversible rubber from supramolecular assembly. Nature 451 (7181), 977–980. 10.1038/nature06669 18288191

[B8] Dimov L. B. Moser M. Malliaras G. G. McCulloch L. (2022). Semiconducting polymers for neural applications. Chem. Rev. 122, 4356–4396. 10.1021/acs.chemrev.1c00685 35089012 PMC9007464

[B9] Etiwy M. Akhrass Z. Gillinov L. Alashi A. Wang R. Blackburn G. (2019). Accuracy of wearable heart rate monitors in cardiac rehabilitation. Cardiovasc. Diagnosis Ther. 9 (3), 262–271. 10.21037/cdt.2019.04.08 PMC6603497 31275816

[B10] Galuska L. A. McNutt W. W. Qian Z. Zhang S. Weller D. W. Dhakal S. (2020). Impact of backbone rigidity on the thermomechanical properties of semiconducting polymers with conjugation break spacers. Macromolecules 53, 6032–6042. 10.1021/acs.macromol.0c00889

[B11] Gruverman A. Yang Y. Huang J. S. Yuan Y. B. Reece T. J. Sharma P. (2011). Efficiency enhancement in organic solar cells with ferroelectric polymers. Nat. Mater. 10 (4), 296–302. 10.1038/nmat2951 21317902

[B12] Hakan U. Chad R. Zhiming W. Hui W. Murat K. D. Aleksandr Z. (2009). Design, synthesis, and characterization of ladder-type molecules and polymers. Air-stable, solution-processable n-channel and ambipolar semiconductors for thin-film transistors via experiment and theory. J. Am. Chem. Soc. 15, 5586–5608. 10.1021/ja809555c 19331320

[B13] Hammock M. L. Chortos A. Tee B. C.-K. Tok J. B.-H. Bao Z. N. (2013). 25th anniversary article: The evolution of electronic skin (E-Skin): A brief history, design considerations, and recent progress. Adv. Mater. 25 (42), 5997–6038. 10.1002/adma.201302240 24151185

[B14] He D. W. Zhang Y. H. Wu Q. S. Xu R. Nan H. Y. Liu J. F. (2014). Two-dimensional quasi-freestanding molecular crystals for high-performance organic field-effect transistors. Nat. Commun. 5, 5162. 10.1038/ncomms6162 25330787

[B15] Hideaki E. Takafumi I. Eigo M. Kazuo T. Masaaki I. Hirokazu K. (2007). Highly soluble [1] benzothieno[3,2-b]benzothiophene (BTBT) derivatives for high-performance, solution-processed organic field-effect transistors. J. Am. Chem. Soc. 51, 15732–15733. 10.1021/ja074841i 18044892

[B16] Hsin-Chiao T. Yen-Wen H. Yu-Cheng C. Yu-Hsuan C. Chu-Chen C. Wen-Ya L. (2021). Intrinsically stretchable polymer semiconductors: Molecular design, processing and device applications. J. Mater. Chem. C 9, 2660–2684. 10.1039/d0tc06059c

[B17] Jeong G. S. Baek D-H. Jung H. C. Song J. H. Moon J. H. Hong S. W. (2012). Solderable and electroplatable flexible electronic circuit on a porous stretchable elastomer. Nat. Commun. 3, 977. 10.1038/ncomms1980 22864570

[B18] Jeong-Il P. Jong W. C. Joo-Young K. Jiyoul L. Ji Y. J. Bonwon K. (2015). Dibenzothiopheno[6,5-b:6',5'-f]thieno[3,2-b]thiophene (DBTTT): High-performance small-molecule organic semiconductor for field-effect transistors. J. Am. Chem. Soc. 38, 12175–12178. 10.1021/jacs.5b01108 25826228

[B19] Kang J. Mun J. Zheng Y. Koizumi M. Matsuhisa N. Wu H.-C. (2022). Tough-interface-enabled stretchable electronics using non-stretchable polymer semiconductors and conductors. Nat. Nanotechnol. 17, 1265–1271. 10.1038/s41565-022-01246-6 36357793

[B20] Kazuki N. Shoji S. Itaru O. Eigo M. Kazuo T. (2011). Dianthra[2,3-b:20,30-f]thieno[3,2-b]thiophene (DATT): Synthesis, characterization, and FET characteristics of new π-extended heteroarene with eight fused aromatic rings. J. Am. Chem. Soc. 22, 8732–8739. 10.1021/ja202377m 21528919

[B21] Kim D-H. Lee Y. (2015). Bioelectronics: Injection and unfolding. Nat. Nanotechnol. 10 (7), 570–571. 10.1038/nnano.2015.129 26053996

[B22] Kim D. H. Lu N. S. Ma R. Kim Y. S. Kim R. H. Wang S. D. (2011). Epidermal electronics. Science 333, 838–843. 10.1126/science.1206157 21836009

[B23] Kim J. Lee M. Shim H. J. Ghaffari R. Cho H. R. Son D. H. (2014). Stretchable silicon nanoribbon electronics for skin prosthesis. Nat. Commun. 5, 5747. 10.1038/ncomms6747 25490072

[B24] Kuribara K. Wang H. Uchiyama N. Fukuda K. Yokota T. Zschieschang U. (2012). Organic transistors with high thermal stability for medical applications. Nat. Commun. 3, 723. 10.1038/ncomms1721 22395614

[B25] Larson C. Peele B. Li S. Robinson S. Totaro M. Beccai L. (2016). Highly stretchable electroluminescent skin for optical signaling and tactile sensing. Science 351 (6277), 1071–1074. 10.1126/science.aac5082 26941316

[B26] Li Y. Chen S. Wu M. Sun J. (2012). Polyelectrolyte multilayers impart healability to highly electrically conductive films. Adv. Mater. 24 (33), 4578–4582. 10.1002/adma.201201306 22807199

[B27] Liang G. Stephen P. D. (2010). High-density stretchable electronics: Toward an integrated multilayer composite. Adv. Mater. 22 (36), 4030–4033. 10.1002/adma.201000515 20717983 PMC3271703

[B28] Liu D. Y. Mun J. Chen G. Schuster N. J. Wang W. C. Zheng Y. (2021). A design strategy for intrinsically stretchable high-performance polymer semiconductors: Incorporating conjugated rigid fused-rings with bulky side groups. J. Am. Chem. Soc. 143, 11679–11689. 10.1021/jacs.1c04984 34284578

[B29] Mei J. G. (2022). What's next for semiconducting polymers. J. Polym. Sci. 60, 287–289. 10.1002/pol.20220014

[B30] Meijer E. J. De Leeuw D. M. Setayesh S. Veenendaal E. V. Huisman B.-H. Blom P. W. M. (2003). Solution-processed ambipolar organic field-effect transistors and inverters. Nat. Mater. 2 (10), 678–682. 10.1038/nmat978 14502272

[B31] Minev I. R. Musienko P. Hirsch A. Barraud Q. Wenger N. Moraud E. M. (2015). Electronic dura mater for long-term multimodal neural interfaces. Science 347 (6218), 159–163. 10.1126/science.1260318 25574019

[B32] Muccini M. (2006). A bright future for organic field-effect transistors. Nat. Mater. 5 (8), 605–613. 10.1038/nmat1699 16880804

[B33] Mun J. Ochiai Y. Wang W. Zheng Y. Zheng Y.-Q. Wu H.-C. (2021). A design strategy for high mobility stretchable polymer semiconductors. Nat. Commun. 12 (1), 3572. 10.1038/s41467-021-23798-2 34117254 PMC8196107

[B55] Odom S. A. Caruso M. M. Finke A. D. Prokup A. M. Ritchey J. A. Leonard J. H. (2010). Restoration of Conductivity with TTF-TCNQ Charge-Transfer Salts. Adv. Funct. Mater 20 (11), 1721 10.1002/adfm.201000159

[B34] Oh J. Y. Rondeau-Gagné S. Chiu Y.-C. Chortos A. Lissel F. Wang G.-J. (2016). Intrinsically stretchable and healable semiconducting polymer for organic transistors. Nature 539 (7629), 411–415. 10.1038/nature20102 27853213

[B35] Okamoto T. Yu C. P. Mitsui C. Yamagishi M. Ishii H. Takeya J. (2020). Bent-shaped p-type small molecule organic semiconductors: A molecular design strategy for next-generation practical applications. J. Am. Chem. Soc. 20, 9083–9096. 10.1021/jacs.9b10450 32293879

[B36] Payne M. M. Parkin S. R. Anthony J. E. Kuo C-C. Jackson T. N. (2005). Organic field-effect transistors from solution-deposited functionalized acenes with mobilities as high as 1 cm^2^/V·s. J. Am. Chem. Soc. 127 (14), 4986–4987. 10.1021/ja042353u 15810810

[B37] Savagatrup S. Zhao X. Chan E. Mei J. Lipomi D. J. (2016). Effect of broken conjugation on the stretchability of semiconducting polymers. Macromol. Rapid Commun. 37 (19), 1623–1628. 10.1002/marc.201600377 27529823

[B38] Shcherbina A. Mattsson C. Waggott D. Salisbury H. Christle J. Hastie T. (2017). Accuracy in wrist-worn, sensor-based measurements of heart rate and energy expenditure in a diverse cohort. J. Personalized Med. 7 (2), 3. 10.3390/jpm7020003 PMC5491979 28538708

[B39] Siebbeles L. D. A. (2010). Two electrons from one photon. Nat. Chem. 2, 608–609. 10.1038/nchem.720 20651716

[B40] Son D. H. Lee J. Qiao S. Ghaffari R. Kim J. Lee J. E. (2014). Multifunctional wearable devices for diagnosis and therapy of movement disorders. Nat. Nanotechnol. 9 (5), 397–404. 10.1038/nnano.2014.38 24681776

[B41] Toan V. P. Jonathan D. Y. Joshua A. K. Braden G. S. Maosheng M. Wesley T. W. (2012). N-Alkyldinaphthocarbazoles,Azaheptacenes, for solution-processed organic field-effect tran-sistors. J. Am. Chem. Soc. 44, 18185–18188. 10.1021/ja3082582 23072644

[B42] Tumbleston J. R. Collins B. A. Yang L. Q. Stuart A. C. Gann E. Ma W. (2014). The influence of molecular orientation on organic bulk heterojunction solar cells. Nat. Photonics 8 (5), 385–391. 10.1038/nphoton.2014.55

[B43] Vissenberg M. C. J M. Matters M. (1998). Theory of the field-effect mobility in amorphous organic transistors. Phys. Rev. B 57 (20), 12964–12967. 10.1103/PhysRevB.57.12964

[B44] Wang C. l. Dong H. Hu W. P. Liu Y. Q. Zhu D. B. (2012). Semiconducting π-conjugated systems in field-effect transistors: A material odyssey of organic electronics. Chem. Rev. 112 (4), 2208–2267. 10.1021/cr100380z 22111507

[B45] Wang Y. Zhu C. Pfattner R. Yan H. Jin L. Chen S. (2017). A highly stretchable, transparent, and conductive polymer. Sci. Adv. 3 (3), e1602076. 10.1126/sciadv.1602076 28345040 PMC5345924

[B46] Wei X. Kristin W. Yanfei W. Roger H. Kazuo T. Bertram B. (2013). Temperature-independent transport in high-mobility dinaphtho-thieno-thiophene (DNTT) single crystal transistors. Adv. Mater. 25, 3478–3484. 10.1002/adma.201300886 23696267

[B47] Williams K. A. Boydston A. J. Bielawski C. W. (2007). Towards electrically conductive, self-healing materials. J. R. Soc. Interface 4 (13), 359–362. 10.1098/rsif.2006.0202 17251165 PMC2359849

[B48] Xie K. Y. Wei B. Q. (2014). Materials and structures for stretchable energy storage and conversion devices. Adv. Mater. 26 (22), 3592–3617. 10.1002/adma.201305919 24643976

[B49] Yamamoto A. Murata Y. Mitsui C. Ishii H. Yamagishi M. Yano M. (2017). Zigzag-elongated fused π-electronic core: A molecular design strategy to maximize charge-carrier mobility. Adv. Sci. 5 (1), 1700317. 10.1002/advs.201700317 PMC5770660 29375963

[B50] Ye G. Song Z. Yu T. Tan Q. Zhang Y. Chen Ti. (2019). Dynamic Ag-N bond enhanced stretchable conductor for transparent and self-healing electronic skin. ACS Appl. Mater. Interfaces 12, 1486–1494. 10.1021/acsami.9b17354 31793286

[B51] Zhang W. Smith J. Watkins S. E. Gysel R. McGehee M. Salleo A. (2010). Indacenodithiophene semiconducting polymers for high-performance, air-stable transistors. J. Am. Chem. Soc. 132 (33), 11437–11439. 10.1021/ja1049324 20677750

[B52] Zhang Y. Basel T. P. Gautam B. R. Yang X. M. Mascaro D. J. Liu F. (2012). Spin-enhanced organic bulk heterojunction photovoltaic solar cells. Nat. Commun. 3, 1043. 10.1038/ncomms2057 22948825

[B53] Zhao Y. Zhao X. Roders M. Qu G. Diao Y. Ayzner A. L. (2015). Complementary semiconducting polymer blends for efficient charge transport. Chem. Mater. 27 (20), 7164–7170. 10.1021/acs.chemmater.5b03349

[B54] Zheng Y. Ashizawa M. Zhang S. Kang J. Nikzad S. Yu Z. (2020). Tuning the mechanical properties of a polymer semiconductor by modulating hydrogen bonding interactions. Chem. Mater. 32, 5700–5714. 10.1021/acs.chemmater.0c01437

